# Direct *in vivo* evidence of immense stem water exploitation in irrigated date palms

**DOI:** 10.1093/jxb/eru421

**Published:** 2014-10-21

**Authors:** Or Sperling, Or Shapira, Amnon Schwartz, Naftali Lazarovitch

**Affiliations:** ^1^The Wyler Department of Dryland Agriculture, French Associates Institute for Agriculture and Biotechnology of Drylands, J. Blaustein Institutes for Desert Research, Ben-Gurion University of the Negev, Israel; ^2^Faculty of Agricultural, Food and Environmental Sciences, the Hebrew University of Jerusalem, Rehovot 76100, Israel; ^3^Northern R&D, Migal, Israel

**Keywords:** Flow, *in vivo*, irrigation, stem, transpiration, water.

## Abstract

During the summer, evaporative demand at midday often exceeds the transport capacity of most desert plants. However, date palms maintain their ecological dominance with sustained and uniquely high rates of transpiration. This high rate of flow cannot be attributed to soil water supply alone. In order to quantify intra-plant water allocation in irrigated date palms, three water-sensing techniques have been incorporated: heat dissipation, gravimetric sampling, and time domain reflectrometry. Each of these methods has known limitations but their integration resulted in a quantitative *in vivo* accounting of the date palm diurnal and seasonal water mass balance. By incorporating these methods it was possible to determine that date palms substantially rely on the exploitation and recharge of the stem reservoir in their water budget. The stem of mature date palms can hold up to 1 m^3^ of water and supply 25% of daily transpiration (i.e. 5000 l of water in 100 d of summer). The internal stem water reservoir is consistently recharged by over 50 l per night which allows for successive daytime reuse throughout the entire growing season. More broadly, these findings suggest that internal water allocation and night-time soil-water availability could provide useful information for improving date palm irrigation practices.

## Highlight

Spatial variations in flow rates and temporal changes in water content show that date palms utilize stem water for daily transpiration and nightly xylem reservoir recharge.

## Introduction

Soil is commonly credited as the main source of water for transpiration in the seasonal ‘whole tree’ mass balance of woody plants (Tyree and Yang, 1990). Nevertheless, temporal intra-system flows play an important role in plant function under transient water conditions. The diurnal depletion of internal water storage allows giant rosettes (*Espeletia*) to transpire when soil water is frozen (Goldstein, 1984), ensures water availability in mature tree canopies at great heights (Waring and Running, 1978; Phillips *et al.*, 2003; Cermák *et al.*, 2007), and compensates for the inability of tree roots to meet midday evaporative demand in the savanna (Scholz *et al.*, 2007). The seasonal exploitation of internal water storage enables the species of arid environments, such as palms or succulents, to withstand drought periods (Smith *et al.*, 1987; Holbrook and Sinclair, 1992b). Thus, when available, an internal water supply ensures optimum physiological activity and enhances stress tolerance.

Common research practices for measuring water flow in plant systems (i.e. lysimeters, sap flow, Bowen ratio, or eddy covariance) cannot reveal variations in water storage as they only detect inter-flows (the flow between a system and its environment). Investigating the intra-system flow requires the evaluation of water status (Waring, 1979; Tyree and Yang, 1990; Scholz *et al.*, 2007; Turcotte *et al.*, 2011), detection of spatial and temporal differences in flow rates (Phillips *et al.*, 2003; Cermák *et al.*, 2007), explicit tracing of water by stable isotopes (James *et al.*, 2003), or the elimination of an external water driving force (i.e. root water uptake or transpiration). Stem water is often measured by destructive gravimetric (Waring, 1979) or water potential (Goldstein, 1984) methods. Such methods, however, are discrete, time consuming, and limited by leaf and stem tissue availability. Alternatively, indirect, non-destructive, and continuous methods have been developed for stem water sensing: e.g. stem dendrometers (Waring and Running, 1978; Cermák *et al.*, 2007) and electrical conductivity (Holbrook *et al.*, 1992; Wullschleger *et al.*, 1996; Nadler and Raveh, 2003). These methods are well-established but they require empirical calibration. Inner-stem flows are commonly measured according to heat pulse, heat dissipation, or heat balance methods. A single sap flow measurement cannot on its own represent variations in water storage but a comparison between flow rates at different locations (e.g. stem and branches) can unveil the displacement of internal water. Finally, the complete detachment of a plant from its water source, as demonstrated by Holbrook and Sinclair (1992b) with palm trees, forces the complete utilization of available water storage. All of these methods noted, there is no comprehensive method among them which is able to produce a total accounting of the water mass balance in trees when stored water is intra allocated.

Date palms (*Phoenix dactylifera* L. Arecaceae) originated in the desert oases of Iraq and adapted to desert conditions and prolonged drought periods. Palms are monocotyledonous with no vascular cambium and, therefore, are permanently dependent on the primary xylem (Tomlinson, 1961; Zimmermann and Tommlinson, 1972) and are ulnerable to cavitation (Zimmermann and Sperry, 1983). A possible protection from deactivated vessels in date palms is that the vascular bundles are uniformly embedded in a ground parenchyma tissue. The live cells have a high specific water capacitance (i.e. ratio between relative water content and the water potential of the tissue) and contain large intercellular air spaces (air volume is enlarged by transpiration-induced tension). Thus, the parenchyma cells can deliver water to xylem vessels and maintain high water potentials (Parthsarathy and Klotz, 1976) with no mechanical stresses (Holbrook *et al.*, 1992; Holbrook and Sinclair, 1992b). Parenchyma cells in palms are more abundant in the stem centre and lower compartments (Holbrook and Sinclair, 1992b) and can support the elevated sap flux densities found in the inner stem (Sellami and Sifaoui, 2003; Sperling *et al.*, 2012).

Hence, defining the diurnal water-storage dynamics may have applied implications for the irrigation regimes of date palm plantations, increasing both water-use efficiency and productivity. Under modern agricultural practices date palms are supplied with large amounts of irrigation water (e.g. 2000mm yr^–1^ in the southern desert of Israel) to achieve optimal plant growth and yield (Tripler *et al.*, 2011). In fact, the soil in date plantations is wetted continuously throughout the main growing season (April–October) and even then field capacity is not usually attained in the entire root zone. Hence, the soil-water supply to transpiration is sometimes insufficient and an alternative reservoir must be required to meet evaporative demand and avoid stomatal closure. Despite a hydraulic bottle-neck at the point of leaf insertion into the stem (Holbrook and Sinclair, 1992a, *b*), irrigated date palms do not withhold transpiration (Tripler *et al.*, 2011). If water consumption is limited, however, tree growth and yields are significantly reduced (Alrasbi *et al.*, 2010; Tripler *et al.*, 2011). Hence, the internal water of date palms plays an important role in maximizing productivity and should be accounted for in irrigation management.

The function of water storage in forest palms has previously been addressed (Holbrook and Sinclair, 1992a, *b*). Yet cultivated palms (e.g. date and oil palms), for which water efficiency and environmental constraints are vital, were overlooked. Moreover, the temporal implications of extreme evaporative demands have not been discussed and direct quantitative evidence of stem water use in irrigated trees is absent. In this study, the aim was to close this knowledge gap by testing the hypothesis that ‘*evaporative demand affects internal water allocation in date palms causing diurnal changes in stem water content’*. Three water-sensing techniques have been incorporated with the objectives of (i) investigating temporal variations in the water storage of field-cultivated date palms; (ii) quantifying the internal water availability of date palms; and (iii) describing seasonal changes in internal water use in date palms.

## Materials and methods

### Experimental site

The research was conducted in a date palm plantation located in the northern Jordan valley (Kibbutz Ashdot Yaakov, 32°39’ N, 35°34’ E), Israel, between June and December, 2012. The Jordan valley region is semi-arid with an annual precipitation of 383mm (December–March). Minimum and maximum daily temperatures are 8.5 °C (January) and 37.7 °C (July), respectively, and the mean daily relative humidity (RH) varies between 75% (January) and 53% (July). The experimental site has mineral alluvial soil and is located at 200 m below sea level. Five date palm trees (*Phoenix dactylifera* L., cv. Hayany) were selected for the research. The trees were 35 years old, 15 m high, and 0.4 m in diameter and were calculated to hold over 1 m^3^ of water [volume multiplied by 60% water content (*WC*)]. The plantation was irrigated through a drip irrigation system (8.0 l h^–1^ drippers, Netafim, Tel Aviv, Israel) following the local field-service extension recommendations (commonly used by growers): multiplying the reference evapotranspiration (ET_0_) by crop and season factors.

### Meteorological data

Environmental conditions were monitored by an *in situ* meteorological station retrieving data on solar radiation (*R*_S,_ MJ m^–2^ d^–1^), wind speed (*U*, m s^–1^), air dry temperature (*T*_air_, ^o^C), and air relative humidity (*RH*, %). Air water vapour pressure deficit (*VPD*, kPa) was calculated from the daily and hourly averages of temperatures and relative humidity. The reference evapotranspiration (*ET*_0_, mm d^–1^) was computed according to the Penman–Montieth (PM) equation (Monteith, 1965) as specified by the FAO Protocol (Allen *et al.*, 1998):

$$ET_{0}=\frac{0.408\Delta \left(R_{\text{n}}-G\right)+\gamma \frac{900}{T+273}U_{2}\left(e_{\text{s}}-e_{\text{a}}\right)}{\Delta +\gamma \left(1+0.34U_{2}\right)}$$ (1)

where Δ (kPa °C^–1^) is the vapour pressure curve slope, *R*_n_ (MJ m^–2^ d^–1^) is the net radiation [derived from solar radiation *R*_s_ (MJ m^–2^ d^–1^)_,_ (Allen *et al.*, 1998)], *G* (MJ m^–2^ d^–1^) is the soil heat flux, γ (kPa °C^–1^) is the psychometric constant, *U*_2_ (m s^–1^) is the wind speed (2 m height), *e*_s_–*e*_a_ (kPa) is the saturation vapour pressure deficit, and *T* (°C) is the air temperature (2 m height).

### Sap flow rate

Four heat dissipation (Granier, 1985) sensors specific for date palms were installed in four trees in May 2012. Each sensor consisted of two identical probes, a heated probe and a reference probe. The probes met the requirements of measuring sap flow (*SF*, l h^–1^ tree^–1^) in mature date palm trees as they were long (33cm) and tough (1cm diameter tubes filled with hardened epoxy glue). The sensors were able to penetrate outer fibrous tissues into the inner stem and measure the surroundings of the thermally conductive tip (2cm long and made of stainless steel, Sperling *et al.*, 2012). The heat dissipation factor, *k* (–), was derived from the maximum (*∆T*_max_, °C) and momentary (∆*T*, °C) temperature differences:

$$k=\frac{\Delta T_{\text{max}}-\Delta T}{\Delta T}$$ (2)

∆*T*_max_ was determined for each sensor during a period of minimal annual transpiration, a winter night, which was set as the reference for zero flow.

*SF* was calculated according to Renninger’s calibration (Renninger *et al.*, 2010), recently reconfirmed for date palms by Sperling *et al.* (2012):

$$SF=192.3k^{1.3}\times A_{\text{eff}}$$ (3)

where *A*_eff_ (–) is the effective section area for water flow and the hourly values were summed for daily total volume flows. Sensors were installed in the centre of the stem (20cm deep) at two heights: 2 m and 10 m above the ground (i.e. 5 m below the canopy). *SF* sensors were hammered into the water-conducting tissues at the centre of the stem through a pre-drilled 8mm hole. A 220V power line reached each experimental unit (tree) and supplied electric power to the sensors and to a datalogger (CR1000, Campbell Scientific, Logan, UT, USA).

### Gravimetric measurements of stem relative water content

Wood samples were taken from four trees at predawn (0500h, which was before transpiration started) and at midday (1200h, when transpiration was at its peak) on 10 August 2012. Horizontal 22cm stem samples were drilled using a 10mm increment borer (Haglof, Langsele, Sweden) at three stem heights: 2, 6, and 10 m above the ground. Samples were weighed, placed in tap water at room temperature for 24h for turgid weight, and oven-dried at 65 °C for 48h for dry weight. The relative water content (*RWC*) was calculated according to:

$$RWC=\frac{W_{\text{f}}-W_{\text{d}}}{W_{\text{t}}-W_{\text{d}}}$$ (4)

where *W*_f_, *W*_d_, and *W*_t_ are the fresh, dry, and turgid weights (g) of the stem samples. The amount of stored water was calculated by multiplying the percentage of stored water by the volume of a mature tree (i.e. 15 m high, 0.4 m in diameter).

### Continuous water content monitoring

A single tree was equipped with six self-manufactured volumetric water-content sensors (September 2012) recording hourly measurements. *WC* was measured at two heights: 2 m and 10 m above the ground (three sensors at each height). Time Domain Reflectometry (TDR) sensors were constructed from two stainless steel rods which were embedded horizontally in the stem 50mm apart (Nadler and Raveh, 2003). The rods (250mm long, 6mm in diameter) reached the centre of the stem and were connected by a 4.9 m coaxial cable (RG58U) to a TDR wave generator (Campbell Scientific, Logan, UT, USA). TDR waveform length was set to 2500mm, starting at the end of the coaxial cable. The bulk dielectric constant (ε_b_) of the stem was derived from the ratio between the electromagnetic (*x*_2_–*x*_1_, m) and the actual (*L*, m) length of the TDR rods:

$$\varepsilon_{\text{b}}={\left(\frac{x_{2}-x_{1}}{V_{\text{p}}L}\right)}^{2}$$ (5)

where *V*_p_ (–) is the relative propagation velocity (commonly set to 0.99). The electromagnetic length of the rod was computed according to the WinTDR (Or *et al.*, 2004) protocol for TDR waveform analysis, where *x*_1_ (m) is the intercept of the two tangent lines at the first maximum point and *x*_2_ (m) is the intercept of two tangent lines at the first minimum. The stem volumetric water content [*WC*, m^3^ (H_2_O) m^–3^] was calculated by a second order quadratic equation (Nadler and Raveh, 2003):

$$WC=-0.251+4.66\times 10^{-2}\varepsilon_{\text{b}}-4.93\times 10^{-2}\varepsilon_{\text{b}}^{2}$$ (6)

The TDR wave generator and datalogger received 12V from a battery which was constantly charged by the 220V power supply.

## Results

**Fig. 1.**
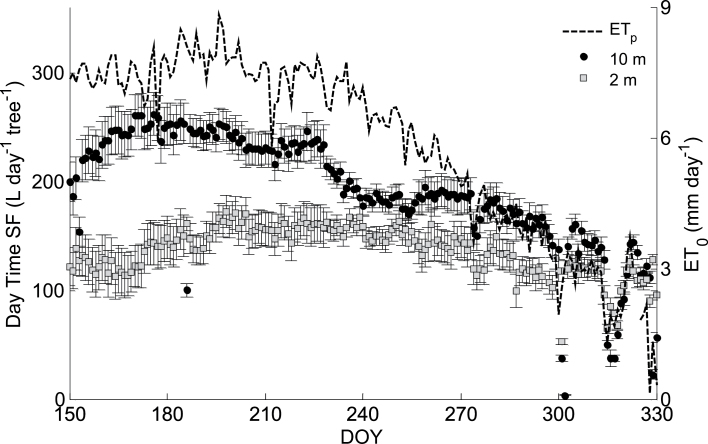
Average (±standard deviation day-time sap flow rates (*SF*) at two stem heights, 2 m (grey squares) and 10 m (black circles), for 200 consecutive days in 2012. Corresponding reference evapotranspiration (*ET*_0_) is also presented (dashed line, right axis).

**Fig. 2.**
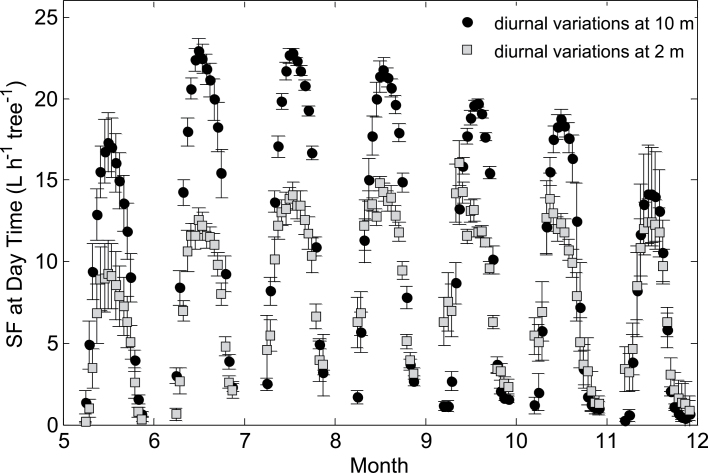
Monthly average (±standard deviation) diurnal sap flow rates (*SF*) at two stem heights, 2 m (grey squares) and 10 m (black circles), for the period of June to November.

*ET*_0_ in the Jordan valley varied between 7.5mm d^–1^ during the summer (June–September) and 2mm d^–1^ during the winter (November–December; Fig. 1). Daytime *SF* in the upper stem (10 m) also decreased from *c*. 250 l d^–1^ tree^–1^ during the summer to *c*. 120 l d^–1^ tree^–1^ during the winter. In late autumn, *ET*_0_ and *SF* varied dramatically across consecutive days due to unstable climatic conditions. However, *SF* in the lower stem (2 m) was limited to 150 l d^–1^ tree^–1^ and was nearly constant throughout both the summer and autumn. *SF* values at 2 m and 10 m were equal only in winter (100 l d^–1^ tree^–1^; Fig. 1). Thus, through the better part of the growing season (June–October), there was an average difference of 56 l d^–1^ tree^–1^ in daytime *SF* between the lower and upper stem. Diurnal measurements reinforced the findings of spatial *SF* variability (Fig. 2). The upper stem *SF* peaked at 22 l h^–1^ tree^–1^ in June and decreased by 25% in November while the lower stem *SF* was nearly constant and reached only 15 l h^–1^ tree^–1^. Apparently, the *SF* at 10 m was analogous to the *ET*_0_ (Fig. 4): it was negligible through the night, increased sharply at 0700h, peaked at noon (*c*. 19 l h^–1^ tree^–1^), and decreased between 1500h and 2000h. Lower-stem *SF* on the other hand increased as early as 0100h, peaked at 1000h (*c*. 14 l h^–1^ tree^–1^), remained constant throughout the afternoon, and approximated the 10 m *SF* only at 1800h (Fig. 4).

**Fig. 3.**
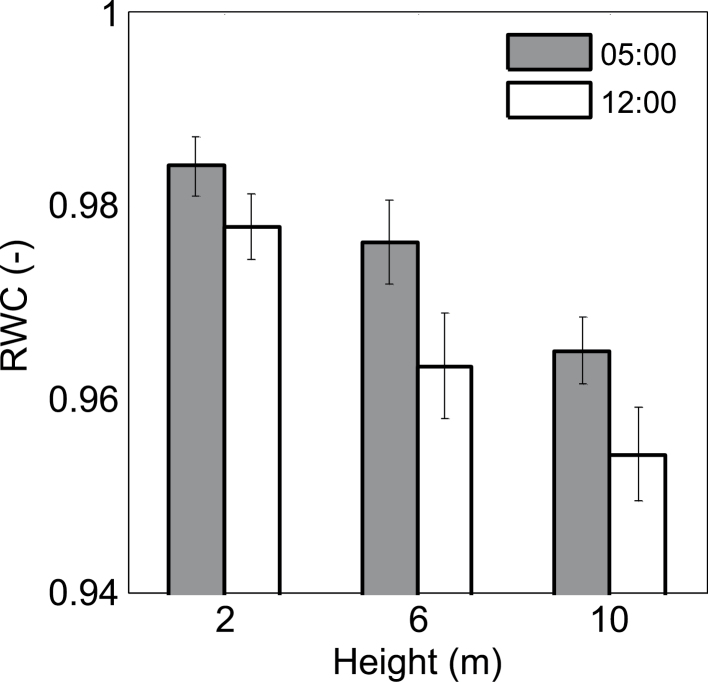
Average (±standard deviation Instantaneous relative water contents (*RWC*) for three stem heights, 2, 6, and 10 m at predawn (0500 h– grey columns) and midday (1200 h– white columns) on 10 August 2012.

**Fig. 4.**
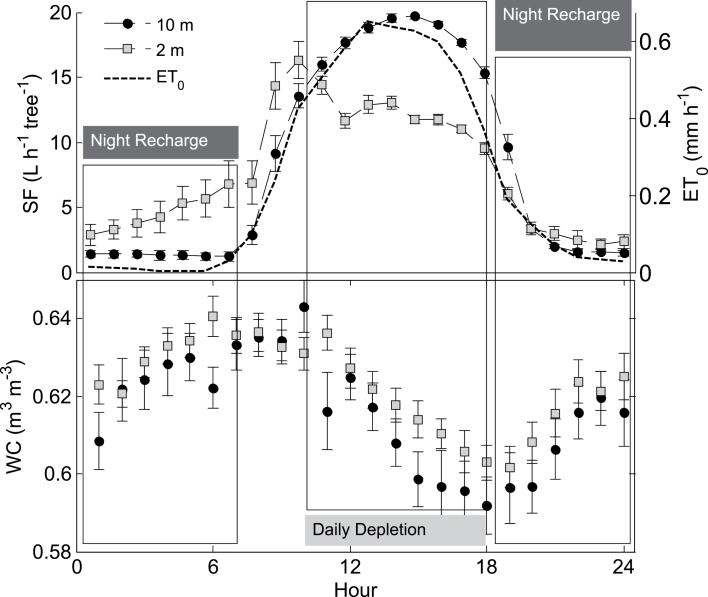
Average (±standard deviation) hourly sap flow rates (*SF*), water content (*WC*), and reference evapotranspiration (*ET*_0_, dashed line) at two stem heights, 2 m (grey squares) and 10 m (black circles), during September 2012. Periods of stem dehydration (noon-evening) and water recharge (night) are labelled.

**Fig. 5.**
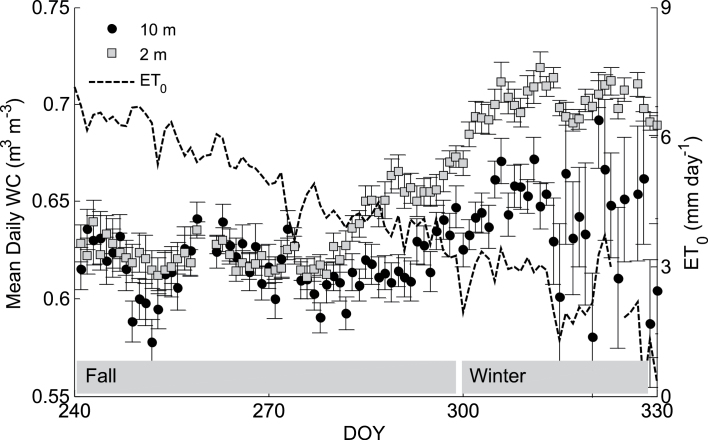
Average (±standard deviation) daily water content at two stem heights, 2 m (grey squares) and 10 m (black circles), for 120 consecutive days in 2012. Corresponding reference evapotranspiration (*ET*_0_) is also presented (dashed line, right axis).

Instantaneous *RWC* of date palm stems was also associated with height (Fig. 3): it was 0.98 at 2 m and 0.95 at 10 m. Furthermore, *RWC* decreased by only 0.5% at 2 m and by over 1% at 10 m between predawn and midday (Fig. 3). Continuous hourly measurements of *WC* further emphasized that the stem has a daily drying and wetting cycle (Fig. 4 shows the average hourly values for September). Stem dehydration occurred between noon (1130h) and late afternoon (1800h) and stem rehydration took place through the night and early morning. The 5% daily difference in *WC* between morning (0700h) and late afternoon (1800h) resulted in a daily depletion of 50 l of stored water from a single tree (Table 1 gives the average diurnal values for September). The stem daily dehydration was compensated for during the night and the average daily *WC* was similar at both heights. Throughout the growing season (June–October) the average *WC* in the stem was 0.62 m^3^ m^–3^ (Fig. 5) and it stored approximately 1 m^3^ of water (for a stem 15 m high and 0.4 m wide; Table 1). In November, *ET*_0_ decreased below 3mm d^–1^_,_ the *SF* at 2 m decreased (Fig. 1), and the lower stem became saturated (*c*. 0.7). However, *WC* at 10 m increased (by 4%) 14 d after the *WC* at 2 m stabilized and it continued to fluctuate according to changes in *ET*_0_.

**Table 1.** Average diurnal differences (Δ*W*) between minimum and maximum values of water content (*WC*) and sap flow rate (*SF*) in September, 2012

	Units	Max	Min	Δ*W* (l d^–1^)
*WC*	m^3^ m^–3^	0.64±0.006	0.59±0.01	55.6
*SF*	l d^–1^	180±8	130±14	50

## Discussion

Peak summer *ET*_0_ is well met by daytime *SF* in the upper stem of date palms (Fig. 1). The transpiration requirements are fulfilled and the physiological performance of the canopy is maintained throughout the main growing season (June–October). At the same time, the evaporative demand at midday in the summer is not fully supplied by the root system, as evidenced by the reduced daytime *SF* in the lower stems (Figs 1 and 2). Evaporative demand may exceed the temporal ability of the root system to supply water due to low soil water availability and limited soil and root hydraulic conductivities (Feddes and Kowalik, 1976). Thus, after ~1030h, an alternative water source is required to supply over 25% of the daily water consumption (Fig. 1; Table 1). This intra-allocation of water would lead to spatial variability of flows. In fact, Goldstein *et al.* (1998) reported that, in tropical forests, basal and canopy sap flows are synchronized only when the internal water reserves are substantially depleted. The findings from the current study point to an internal water allocation which is overlooked by the common soil–plant–atmosphere water mass balance approach. Disregarding this water utilization leads to an underestimate of instantaneous water transport, misleading water stress measurements, and ignorance to the importance of night time irrigation. As palm leaves have a low water content and a low specific capacitance (Holbrook and Sinclair, 1992b) the water reservoir could only be in the stem. There is over 1 m^3^ of water stored in parenchyma tissue (subject to the volume and *WC* of the stem; see the Materials and methods) available for intra-stem allocation under reduced water potentials (Holbrook and Sinclair, 1992b). This water reservoir is effective for more than 6 months of the year (Fig. 2) and during this time it must be refilled on a daily basis. As the trees are well-irrigated, stem-water refilling may take place at night, when transpiration is minimized (Holbrook and Sinclair, 1992a; Goldstein *et al.*, 1998; Cermák *et al.*, 2007; Turcotte *et al.*, 2011). The *SF* heat dissipation sensors are inadequate for detecting flows at night due to prior assumptions of zero night flow and low sensitivity (Granier, 1985; Sperling *et al.*, 2012). This leads to an incomplete water mass balance which neglects the allocation of water within the tree. Nevertheless, if corrected for an annual reference of minimum flow, hourly readings of *SF* reveal high water intake in the lower stem during the night (Fig. 4).

Stem xylem vessels have low specific water capacitance (Scholz *et al.*, 2007). In palms, the upper stem is younger and parenchyma tissue is less lignified, with thinner walls and the potential for induced air spaces (Parthsarathy and Klotz, 1976). Hence, the water storage requirements of the canopy are probably met by the parenchyma of the upper stem (Zweifel *et al.*, 2001). This elastic water storage prevents the over-extraction of xylem water (Tyree and Yang, 1990) and, consequently, protects the stem from embolism. Thus, not only does the stem supply additional water for transpiration, but it is the primary defence against loss of stem hydraulic integrity. This is especially important in the non-renewable xylem vessels of the monocotyledon palms. The palm stem goes through repetitive variations in *WC* while dehydrating and replenishing 5% of its available water (Fig. 4). This change in water storage is very similar to the gap in *SF* (55 versus 50 l d^–1^, respectively; Table 1). This report of 20% stem daily water loss is consistent with previous publications about *arborescent* palms in the greenhouse (Holbrook and Sinclair, 1992a) and Douglas-fir trees in the forest (Cermák *et al.*, 2007). Stem *SF* and *WC* are synchronized in date palms as dehydration begins when lower-stem *SF* falls below upper-stem *SF* (1100h) and ceases when the two flows are back in equilibrium (1700h; Fig. 4). Night-time stem re-watering begins when transpiration ceases completely (2000h) and continues throughout the night until transpiration is reactivated (0600h). This is a clear demonstration of intra-flow of water to the stem and canopy according to temporal requirements.

The hourly variations in water sources for transpiration are seasonal and depend on soil water availability and evaporative demand. Wullschleger *et al.* (1996) reported 40% variations in stem *WC* in red maple trees, peaking in the summer of a wet year and decreasing towards winter. In date palms, the high summer day-time evaporative demand imposes the major use of stem water and differentiates between lower and upper stem flows (Fig. 1). Yet, stem water storage is continuously recharged and the average daily water content is unchanged (Fig. 5). Towards winter the evaporative demand is again met by roots, the flows equilibrate, and stem *WC* increases. At this time of the year the lower stem is nearly saturated as water-storage depletion is limited to the upper stem which is still affected by the evaporative demand.

## Conclusions

Under the dry summer conditions of a Mediterranean climate, the date palm is largely dependent on its stem water reservoir to meet hourly transpiration requirements and maintain diurnal and seasonal homeostasis in water uptake. Due to high intensity irrigation, cultivated date palms do not encounter prolonged water stress. Nevertheless, it was found that the midday evaporative demand during summer exceeds the water supplied by roots and stem water storage is depleted. The internal reservoir contributes 25% of the daily transpiration and is recharged at night for subsequent daytime use. Diurnal intra-water allocation is crucial to the photosynthetic performance of date palms as it enables optimum vegetative and reproductive growth in hot and dry desert summers. This makes the desert-adapted date palm a highly sustainable, productive, and economic crop in arid Mediterranean regions.
